# Polarized running training adapted to versus contrary to the menstrual cycle phases has similar effects on endurance performance and cardiovascular parameters

**DOI:** 10.1007/s00421-024-05545-9

**Published:** 2024-07-08

**Authors:** Claudia Kubica, Sascha Ketelhut, Claudio Renato Nigg

**Affiliations:** Health Science Department, University of Bern, Bern, Switzerland

**Keywords:** Females, Naturally menstruating, Endurance training, $\dot{V}$O_2max_, Hemodynamics

## Abstract

**Purpose:**

This study compared the effects of polarized running training adapted to the menstrual cycle (MC) phases versus polarized training adapted contrary to the MC on endurance performance and cardiovascular parameters.

**Methods:**

Thirty-three naturally menstruating, moderately trained females (age: 26 ± 4 years; BMI: 22.3 ± 3.2 kg/m^2^; $\dot{V}$O_2max/rel_: 40.35 ± 4.61 ml/min/kg) were randomly assigned to a control (CON) and intervention (INT) group. Both groups participated in a load-matched eight-week running training intervention. In the INT, high-intensity sessions were aligned with the mid and late follicular phase, low-intensity sessions with the early and mid-luteal phase, and recovery with the late luteal and early follicular phase. In the CON, high-intensity sessions were matched to the late luteal and early follicular phase, and recovery to the mid and late follicular phase. Endurance performance and cardiovascular parameters were assessed at baseline and after the intervention.

**Results:**

Twenty-six females completed the intervention. A repeated measures ANOVA determined no time × group interaction effect for any parameter. A significant time effect was found for maximal oxygen uptake (*F*(1,12) = 18.753, *p* = 0.005, *η*_p_^2^ = 0.630), the velocity at the ventilatory threshold one (*F*(1,12) = 10.704, *p* = 0.007, *η*_p_^2^ = 0.493) and two (*F*(1,12) = 7.746, *p* = .018, *η*_p_^2^ = .413).

**Conclusion:**

The training intervention improved endurance performance in both groups, with no further benefit observed from the MC-adapted polarized training in a group-based analysis. Replications with an extended intervention period, a larger sample size, and a more reliable MC determination are warranted.

## Introduction

Throughout a female's life, from menarche to the onset of menopause, a regular biological rhythm known as the menstrual cycle (MC) governs the ebb and flow of endogenous sex hormones, particularly estrogen and progesterone (de Jonge et al. 2019). These hormonal fluctuations orchestrate the reproductive system's function while exerting influence over other physiological systems, including the cardiovascular, respiratory, and nervous systems (Bernstein and Behringer 2023).

Variations in the concentrations of these hormones, specifically estrogens and progesterone, may have further implications for athletic performance (McNulty et al. 2020). Even though isolating single actions of hormones due to interdependency is challenging, estrogens and progesterone exhibit distinct influences on the energy metabolism in naturally menstruating females. Estrogens appear to impact the oxidation of energy substrates, leading to an increased rate of carbohydrate oxidation (Zderic et al. 2001) and a higher utilization rate of glycogen oxidation (Devries 2016), ultimately favoring increased endurance performance (Bernstein and Behringer 2023). On the other hand, progesterone, acting as an estrogen antagonist, inhibits carbohydrate oxidation, resulting in increased protein catabolism and higher rates of amino acid oxidation (Boisseau and Isacco 2021). Furthermore, during the luteal phase, which is characterized by elevated progesterone concentrations, an increase in muscle glycogen-sparing effects (Devries 2016; Oosthuyse and Bosch 2010), and an enhanced reliance on lipid metabolism can be observed (Oosthuyse and Bosch 2010; Willett et al. 2021). These metabolic variations may affect individual training readiness and overall training responses throughout the MC.

Even though general guidelines on modulating exercise training according to the MC do not exist, adapting the training to the MC may alter long-term performance development (Recacha-Ponce et al. 2023). While previous research reports a positive impact of adjusting resistance training variables according to the MC on different performance outcomes (Kissow et al. 2022), there remains a notable gap in knowledge regarding endurance training. To our knowledge, only one previous publication from our research group explored this topic (Kubica et al. 2023). In this study, we compared the effects of a traditional eight-week block periodized running training with a polarized running training adapted to the MC phases on a range of variables, including endurance performance, cardiovascular parameters, recovery, and MC-related symptoms. A major limitation of this study was that, although randomly allocated to intervention and control groups, a significant portion of the control group's training coincidentally aligned with the MC phases, leading to only minor differences in the training protocols between the groups. This made it challenging to identify any potential influences of the MC.

Considering the limitations of the previous study and lack of research in the field, the primary study adopts an exploratory approach to compare the effects of polarized training adapted to the MC phases with polarized training explicitly adapted in contrast to the MC phases on endurance performance and cardiovascular parameters in naturally menstruating females. By devising distinct training interventions, we aim to gain a clearer understanding of the impact of MC-adapted running training on endurance performance and cardiovascular outcomes.

## Methods

### Study design and participants

A parallel arm randomized controlled design involving forty moderately active females was implemented. Participants were eligible to take part in the study if they met the following criteria: (1) had a regular menstrual cycle length (between 21 and 35 days with no more than ± 3 days of variation within the last 6 months), (2) were not pregnant (last pregnancy > 1 year), (3) were not using hormonal contraceptives (not within the last 6 months), (4) had no underlying health conditions or orthopedic injuries, (5) exhibited ovulation, and (6) met the minimum physical activity requirements (> 150 min of moderate/vigorous physical activity per week with at least 15 min of vigorous physical activity per week).

Participants were recruited via announcements at the University of Bern, personal contact, and social media posts. The recruitment and data collection period spanned from December 2022 to April 2023.

**Fig. 1:**
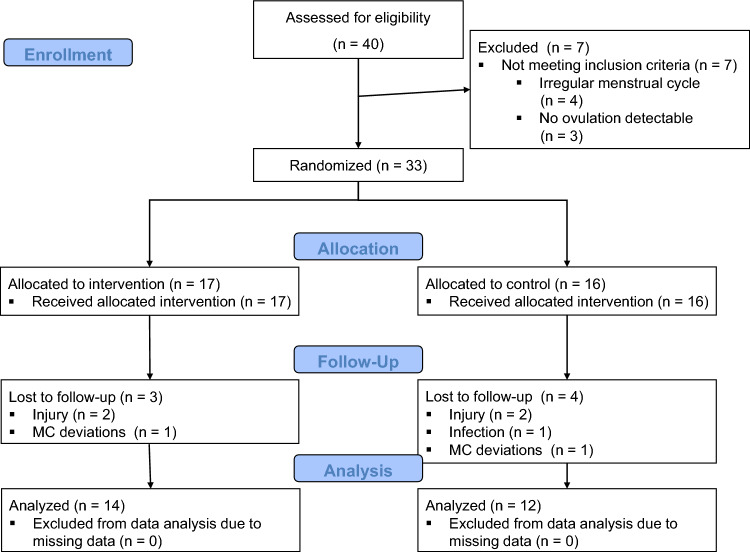
Flowchart

**Fig. 2:**
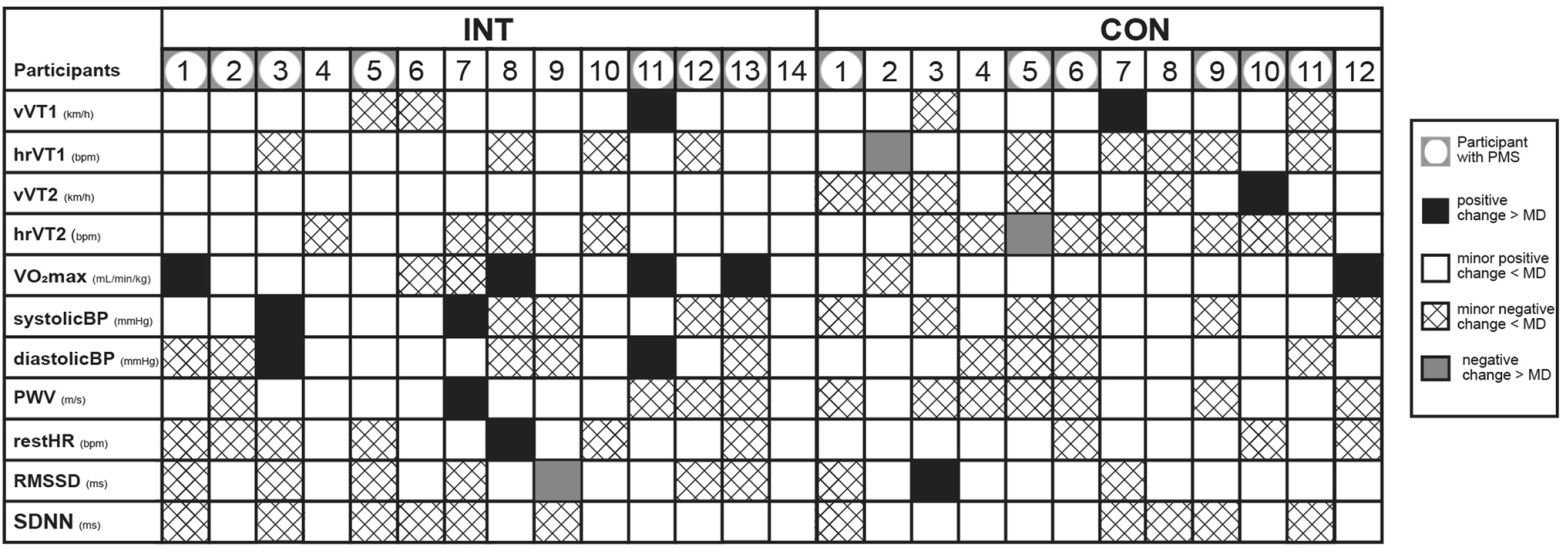
Individual patterns of training responses based on MD. Individual patterns of response following eight weeks of training. Positive responses with an individual change from baseline to post-assessment larger than the MD (black boxes), minor responses smaller than the MD (negative minor change-scattered boxes and positive minor change—white boxes), and adverse responses exceeding the MD (grey boxes) are shown for all participants across all variables. *MD* minimal difference to be considered real, *MD%* percentage of participants with a difference ≥ MD, *INT* intervention group, *CON* control group, *vVT1* velocity at ventilatory threshold 1, *hrVT1* heart rate at ventilatory threshold 1, *vVT2* velocity at ventilatory threshold 2, *hrVT2* heart rate at ventilatory threshold 2, $\dot{V}$*O*_*2max/M*_ maximal oxygen consumption normalized per body mass, *BP* blood pressure, *PWV* pulse wave velocity, *restHR* resting heart rate, *RMSSD* root mean square of successive RR interval differences, *SDNN* Standard Deviation of NN Interval, *PMS* premenstrual Syndrome

Seven participants did not meet the inclusion criteria and were subsequently excluded from the study (Fig. 1 Flow Chart). A total of 33 participants (age: 26 ± 4 years; body mass index (BMI): 22.3 ± 3.2 kg/m^2^) were randomly assigned either to a control group (CON) or an intervention group (INT) (Fig. 2). The principal investigator conducted the randomization using a computer-generated random number table.

Each participant provided written, informed consent after being presented with an explanation of the study's objectives and the experimental procedures. This study received approval from the Ethical Commission of the Faculty of Human Sciences at the University of Bern (Nr. 2022-01-00006).

### Measurements

Participants underwent a baseline assessment, an 8-week intervention, and a post assessment. Both testing sessions occurred at the Institute of Sport Science of the University of Bern on the same time of day at the same day of the week to minimize circadian fluctuations. For each testing session, participants were asked to be at least 2 h postprandial, refrain from consuming caffeine and alcohol for a minimum of 4 h, and avoid exercising for at least 48 h. All measurements were conducted by well-trained research staff using standardized equipment and procedures in controlled conditions. The measurements during the baseline and post-assessments were conducted in the following order: demographics and anthropometrics, cardiovascular parameters, and endurance performance.

### Demographics and anthropometrics

Demographic data, medical history, and premenstrual symptoms were obtained through questionnaires. To assess premenstrual symptoms, the German version of the screening instrument for premenstrual symptoms (SIPS) (Bentz et al. 2012) was administered once during the baseline assessment.

Standing height and body mass were determined using a stadiometer and a body composition scale (RD 545HR, Tanita Europe BV, Amsterdam, Netherlands). Waist circumference was measured with non-elastic tape at the midpoint between the costal arch and the upper edge of the iliac crest. Body Mass Index (BMI) was then computed based on weight in kilograms and height in meters (kg/m^2^). The Waist-to-Height Ratio (WHtR) was determined by dividing waist circumference by height (waist circumference/height).

### Endurance performance

A graded exercise test on a treadmill ergometer (h/p/cosmos pulsar 4.0; h/p/cosmos sports and medical GmbH, Nussdorf-Traunstein, Germany) was conducted to assess maximal oxygen consumption ($\dot{V}$O_2max_). The test started with an initial speed of 6.6 km h^−1^, and increased stepwise by 1.2 km h^−1^ every 3 min, with 30 s of passive rest after each step until volitional exhaustion was achieved.

Oxygen consumption was continuously monitored using a breath-by-breath gas analyser (Metalyzer 3B, Cortex, Leipzig, Germany). $\dot{V}$O_2max_ was computed as the highest recorded value, using a rolling average of 15-s intervals. To ensure the accuracy of the measurements, a two-point calibration procedure was performed prior to each test day. This procedure involved calibrating the oxygen and carbon dioxide sensors with gases of known concentrations and calibrating the flow rate using a 3-L syringe. Additionally, ambient air calibration was conducted before each test.

To confirm the attainment of $\dot{V}$O_2max_, at least three of the following criteria had to be met: (1) a final rating of perceived exertion score of ≥ 17 on the Borg scale (scale 6–20), (2) a respiratory exchange ratio > 1.1, (3) no further change in heart rate (HR) with an increase in workload, (4) a "plateau" (an increase of ≤ 150 ml) in oxygen uptake with a simultaneous increase in workload, (5) volitional fatigue.

Two investigators independently identified ventilatory thresholds (VT1 and VT2). If there was a lack of agreement, the opinion of a third observer was sought. VT1 was defined as the workload at which increases were observed in the ventilatory equivalent for oxygen and the end-tidal pressure of oxygen without a simultaneous rise in the ventilatory equivalent for carbon dioxide. Similarly, VT2 was determined when increases were evident in the ventilatory equivalent for oxygen and the ventilatory equivalent for carbon dioxide, accompanied by a reduction in the end-tidal pressure of carbon dioxide (Amann et al. 2004; Gaskill et al. 2001).

HR was continuously recorded beat-to-beat throughout the graded exercise test using a Polar HR sensor (H10, Polar Electro Oy, Kempele, Finland).

### Cardiovascular parameters

#### Hemodynamics

Peripheral systolic blood pressure (BP), peripheral diastolic BP, and pulse wave velocity (PWV) were assessed utilizing the Mobil-O-Graph^®^ (PWA-Monitor, IEM, Stollberg, Germany), a clinically validated device (Franssen and Imholz 2010). Two measurements were taken on the upper right arm using customized arm cuffs following 10 min of supine rest. The mean of the two measurements was used for analysis.

#### Heart rate variability (HRV)

Heart rate variability (HRV) was assessed using a HR sensor and a chest strap (Polar RS800 CX^®^, Polar Electro OY, Kempele, Finland). Following 5 min of rest in a supine position, a 5-min measurement was conducted. Throughout the measurement, patients were instructed to maintain a normal and comfortable breathing pattern.

The raw data underwent processing using the app Elite HRV (Elite HRV Inc, 2022), which has demonstrated validity and reliability (Moya-Ramon et al. 2022). The analysis included the assessment of the root mean square of successive RR interval differences (RMSSD), the standard deviation of NN intervals (SDNN), and the resting HR (restHR).

### Determination of the MC and cycle phasing

A month before the intervention, a three-step verification process was employed to confirm that participants met the inclusion and exclusion criteria and to ascertain their MC phases during the intervention. Furthermore, it was examined whether the MC characteristics of the participants aligned with the definition of naturally menstruating females (Elliott-Sale et al. 2021). This three-step verification encompassed the following:

1. Calendar-based counting: Individuals tracked and recorded the onset and duration of their menstrual periods on a calendar, using historical data to calculate the approximate dates for future MC.
2. Measurement of basal body temperature: Each morning, upon waking, participants measured their basal body temperature using the Breuer FT 09 thermometer (Breuer GmbH, Ulm, Germany).
3. Ovulation: Participants were provided with ovulation tests (Pinkline Ovulation Test 25 mlU/mL, Pinkline By Burggraf, Taverne, Switzerland) with the recommended threshold of 25 mlU/mL (Leiva et al. 2017). These kits involved colorimetric enzyme immunoassays of urinary luteinizing hormone. Participants were instructed to conduct the ovulation tests according to the manufacturer's directions. Further, ovulation tests were performed from the seventh day of the MC onwards. Ovulation was assumed to have occurred one day after a positive ovulation test. If no positive ovulation test was recorded during the MC, testing was postponed for one more cycle until a positive ovulation test was observed. Participants were excluded from the study if they experienced two consecutive cycles without a positive ovulation test (s. Fig. 1).

MC phases were calculated according to the recommendation from Schmalenberger et al. (2021) as follows:

- Mid to late follicular phase including periovulation: + 4 days after menstrual onset until periovulation + 1 day following a positive ovulation test and nadir.
- Early luteal–mid-luteal phase: + 2 days following a positive ovulation test and nadir until + 10 days following a positive ovulation test and nadir.
- Luteal phase and menstruation/early follicular phase: − 3 days before onset of bleeding until + 4 days after menstrual onset.

### Training protocol

The training protocol consisted of an 8-week running training intervention, including three weekly training sessions. Both groups followed a general polarized running training program, which is recognized an effective training approach for recreationally active runners (Muñoz et al. 2014). The program was designed to attain a total percentage distribution in training zones 1, 2, and 3 of 75%/5%/20% based on HR distribution and running velocity at the VT1 and VT2. In the INT, the single running training sessions were adapted according to current MC training recommendations (Elliott-Sale and Pitchers 2019). Running training sessions including Zone 3 training, were mainly performed during the mid and late follicular phase, matched with the parallel increase in oestrogen concentration, which presumably improves the oxidation of carbohydrates and the uptake of glycogen into type I muscle fibres (Hackney 2021). Running training sessions, including Zone 1 and 2, were mainly performed during the early and mid-luteal phases, characterized by high levels of progesterone, which is suggested to increase the reliance on fat metabolism (Hackney 2021). During the premenstrual and menstrual phases, only running training sessions, including Zone 1 with a reduced volume, were performed (Elliott-Sale and Pitchers 2019; Recacha-Ponce et al. 2023). The CON followed the same running training program. However, the single running training sessions were contrary adapted to their MC. Running training sessions, including Zone 3 training, were mainly performed during the premenstrual and menstrual phases. Zone 1 running training with a reduced volume was performed during the mid and late follicular phase. Running training during the early and mid-luteal phase was comparable to the INT, with training sessions in Zone 1 and 2. We chose to maintain a consistent training frequency throughout the intervention duration for both the INT and CON groups, while varying the intensity of the training intervention. This decision was made to mitigate the potential risk of injuries associated with fluctuating training frequencies, as suggested by Ferreira et al. (2012).

Both interventions were designed to have equivalent workloads and were HR controlled (H10, Polar Electro OY, Kempele, Finland). Participants were instructed to record their training frequency, volume, and average training HR in a digital diary (m-path application, KU Leuven R&D, Leuven, Belgium). Participants who missed more than three of the 24 training sessions were excluded from the analysis.

### Statistics

We analyzed data using IBM SPSS Statistics for Windows, Version 27.0 (IBM Corp. Released 2020, Armonk, NY, USA). The results are reported as means ± standard deviation. To assess differences in subject characteristics between the groups at baseline, we employed independent samples *t* tests. Repeated measures analysis of variance (ANOVA) was utilized to investigate the interactions between time × group regarding the outcomes. Post-hoc analyses with Bonferroni's correction were conducted if appropriate. ANOVA effect sizes (partial eta squared (*η*_p_^2^)) are defined as small, medium, and large: 0.01 to ≤ 0.06, 0.06 to < 0.14, and ≥ 0.14, respectively (Richardson 2011). The effect size for the *t* tests was measured by Cohen's *d* (*d*). Small, medium, and large effect sizes were designated as ¦*d*¦ = 0.2, ¦*d*¦ = 0.5, and *¦d*¦ = 0.8, respectively (Cohen 2013). Statistical significance was set a priori at *p* < 0.05.

To determine the reliability of the variables, intraclass correlation coefficients (ICC) and their 95% confidence intervals were calculated based on a mean rating, consistent, 2-way mixed-effects model (Koo and Li 2016; Weir 2005). ICC values less than 0.5 indicate poor reliability, values between 0.5 and 0.75 reveal moderate reliability, values between 0.75 and 0.9 reveal good reliability, and values greater than 0.90 reveal excellent reliability (Portney and Watkins 2009). Furthermore, standard error of measurement (SEM) and minimum difference to be considered real (MD) were calculated as follows (Weir 2005):

$$MD=SEM \times 1.96 \times \sqrt{2}.$$

## Results

### Participants' characteristics

No adverse events were recorded during the assessments for any of the patients. Seven participants (INT = 3, CON = 4) were lost before post-assessments due to various reasons, as indicated in Fig. 1. Consequently, fourteen females from the INT (age: 26 ± 4; BMI: 21.7 ± 2.8 kg/m^2^; $\dot{V}$O_2max/*M*_: 39.9 ± 4.6 mL min^−1^ kg^−1^) and twelve females from the CON (age: 26 ± 3; BMI: 22 ± 2.2 kg m^−2^; $\dot{V}$O_2max/*M*_ 40.8 ± 4.8 mL min^−1^ kg^−1^) were incorporated in the final analysis (Table 1). No significant group differences (*p* < 0.05) were identified in the baseline assessment for all parameters.

**Table 1:** Participant's characteristics at baseline assessment

Outcome	Total	INT	CON	*p *value	Effect size *d*
N/%female	26/100	14/100	12/100	–	–
MC length (d)	29.4 ± 2.5	29.4 ± 2.9	29.3 ± 2.0	0.924	0.0.38
Age (year)	25.9 ± 3.5	25.9 ± 4.0	25.9 ± 3.0	0.966	-0.017
Height (m)	1.66 ± 0.05	1.67 ± 0.06	1.66 ± 0.43	0.966	-0.017
Body mass (kg)	60.4 ± 6.1	60.2 ± 5.9	60.6 ± 6.6	0.856	-0.072
BMI (kg (m^2^)^−1^)	21.8 ± 2.5	21.7 ± 2.8	22.0 ± 2.2	0.785	-0.109
Body fat (%)	24.5 ± 5.9	24.1 ± 6.1	25.0 ± 5.9	0.707	-0.154
WHtR	0.42 ± 0.03	0.43 ± 0.04	0.42 ± 0.02	0.522	0.245

Values are calculated with two-tailed independent *t* test. Data are presented as mean ± standard deviation, and *p* values indicate differences between the INT and CON. Effect size are reported as Cohen's *d*.

Based on BMI, two participants from the INT and one from the CON were classified as underweight (Weisell 2002). According to the WHtR cut-off point of 0.5, all participants were within the healthy range and not characterized as central obese (Yoo 2016). Based on peripheral BP results, two participants in the CON and one in the INT were classified as hypertensive (Flack and Adekola 2020). Regarding $\dot{V}$O_2max/*M*_, participants in the CON were categorized as follows: three were untrained, and nine were active. In the INT, six participants were considered untrained, and eight as active (Decroix et al. 2016). According to the SIPS and following the criteria established by Bentz et al. (2012), six participants in the CON and seven in the INT had premenstrual symptoms

*INT* intervention group, *CON* control group, *MC length* average menstrual cycle length in the previous three MCs, *BMI* body mass index, *WHtR* Waist-to-Height-Ratio

### Endurance performance

We found no statistically significant difference in endurance parameters according to the groups over time (vVT1 (*F*(1,12) = 0.153, *p* = 0.703, *η*_p_^2^ = 0.014), hrVT1 (*F*(1,12) = 3.039, *p* = 0.109, *η*_p_^2^ = 0.216), vVT2 (*F*(1,12) = 0.409, *p* = 0.535, *η*_p_^2^ = 0.036), hrVT2 (*F*(1,12) = 2.521, *p* = 0.141, *η*_p_^2^ = 0.186); and $\dot{V}$O_2max/*M*_ (*F*(1,12) = 1.017, *p* = 0.335, *η*_p_^2^ = 0.850) (s. Table 2).

**Table 2:** Endurance performance at baseline and post assessments

Outcome	INT		CON		*p* value (group × time)	*η*_p_^2^ (group × time)	*p *value (time)	*η*_p_^2^ (time)
	Baseline	Post	Baseline	Post				
vVT1 (km h^−1^)	5.1 ± 1.7	6.8 ± 1.2	4.9 ± 1.9	6.4 ± 1.8	0.703	0.014	0.007**	0.493
hrVT1 (b min^−1^)	144.8 ± 11.9	138.9 ± 20.6	136.9 ± 9.9	140.8 ± 6.4	0.109	0.216	0.634	0.021
vVT2 (km h^−1^)	11.2 ± 1.2	11.8 ± 0.9	11.4 ± 1.5	11.7 ± 1.1	0.535	0.036	0.018*	0.413
hrVT2 (b min^−1^)	183.6 ± 9.9	181.8 ± 5.9	178.1 ± 7.7	180.0 ± 5.4	0.141	0.186	0.975	0.000
$\dot{V}$O_2max/*M*_ (mL min^−1^ kg^−1^)	39.25 ± 4.62	43.00 ± 4.47	40.83 ± 4.80	43.25 ± 4.39	0.335	0.85	0.001**	0.630

Data are presented as mean ± standard deviation. Differences between groups and baseline and post-assessment were calculated with a two-factorial ANOVA with repeated measurements. *p* values indicate interaction, group and time effects. Significant differences are highlighted with * and ** (*p < 0.05, **p < 0.01). Effect size is reported as *η*_p_^2^

*INT* intervention group, *CON* control group, *vVT1* velocity at ventilatory threshold 1, *hrVT1* heart rate at ventilatory threshold 1, *vVT2* velocity at ventilatory threshold 2, *hrVT2* heart rate at ventilatory threshold 2, $\dot{V}$*O*_*2max/M*_ maximal oxygen consumption normalized per body mass

However, a significant time effect was found for $\dot{V}$O_2max/*M*_ (*F*(1,12) = 18.753, *p* = 0.005, *η*_p_^2^ = 0.630), vVT1 (*F*(1,12) = 10.704, *p* = 0.007, *η*_p_^2^ = 0.493) and vVT2 (*F*(1,12) = 7.746, *p* = 0.018, *η*_p_^2^ = 0.413), but not for the other endurance parameters. No group effects were found for any of the endurance parameters

### Cardiovascular parameters

No significant time × group interactions effects were found for the cardiovascular parameters (systolic BP (*F*(1,12) = 2.092, *p* = 0.176, *η*_p_^2^ = 0.160), diastolic BP (*F*(1,12) = 0.144, *p* = 0.711, *η*_p_^2^ = 0.013), restHR (*F*(1,12) = 0.001, *p* = 0.979, *η*_p_^2^ = 0.000), RMSSD (*F*(1,12) = 2.617, *p* = 0.134, *η*_p_^2^ = 0.192) and SDNN (*F*(1,12) = 1.122, *p* = 0.312, *η*_p_^2^ = 0.093)) (Table 3). Additionally, no significant time effects were found for any of the cardiovascular parameters.

**Table 3:** Cardiovascular parameters at baseline and post assessments

Outcome	INT		CON		*p* value (group × time)	*η*_p_^2^ (group × time)	*p *value (time)	*η*_p_^2^ (time)
	Baseline	Post	Baseline	Post				
Systolic BP (mmHg)	115.5 ± 10.8	109.3 ± 8.1	118.7 ± 12.1	117.8 ± 9.5	0.176	0.160	0.184	0.155
Diastolic BP (mmHg)	69.0 ± 6.9	66.9 ± 6.8	71.3 ± 8.6	70.4 ± 6.0	0.711	0.013	0.228	0.129
PWV (m s^−1^)	5.06 ± 0.31	4.87 ± 0.33	5.10 ± 0.39	5.10 ± 0.30	0.211	0.138	0.161	0.170
restHR (b min^−1^)	72.7 ± 12.8	68.9 ± 14.2	71.3 ± 9.1	67.6 ± 7.9	0.979	0.000	0.760	0.259
RMSSD (ms)	56.67 ± 37.17	54.72 ± 26.27	51.95 ± 27.20	67.70 ± 35.95	0.134	0.192	0.223	0.192
SDNN (ms)	69.08 ± 33.60	64.89 ± 20.50	73.45 ± 27.67	79.07 ± 32.88	0.312	0.093	0.901	0.001

Data are presented as mean ± standard deviation. Differences between groups and baseline and post-assessment were calculated with a two-factorial ANOVA with repeated measurements. *p *values indicate interaction, group and time effects. Significant differences are highlighted with * and ** (*p < 0.05, **p < 0.01). Effect size is reported as *η*_p_^2^

*INT* intervention group, *CON* control group, *sBP* systolic blood pressure, *dBP* diastolic blood pressure, *PWV* pulse wave velocity, *restHR* resting heart rate, *RMSSD* Root mean square of successive RR interval differences, *SDNN* Standard Deviation of NN Interval

### ICC, SEM and MD

The ICC, SEM, MD, and the proportion of participants with changes exceeding the MD are reported in Table 4. The outcomes showed poor reliability for vVT1, systolic BP, diastolic BP, and PWV, and moderate reliability for hrVT1, vVT2, hrVT2, $\dot{V}$O_2max*/M*_, restHR, RMSSD and SDNN. Based on the ICC and SEM, the following MD resulted for the performance parameters: vVT1: 4.2 km h^−1^, hrVT1: 21.5 b min^−1^, vVT2: 1.7 km h^−1^, hrVT2: 14.2 m min^−1^, $\dot{V}$O_2max*/M*_: 7.0 mL min^−1^ kg^−1^; and for the cardiovascular parameters: systolic BP: 21.5 mmHg, diastolic BP: 12.2 mmHg, PWV: 0.7 m s^−1^, restHR: 16.5 b min^−1^, RMSSD: 7.0 ms, and SDNN: 230 ms.

**Table 4:** ICC, SEM and MD for endurance and cardiovascular parameters

Outcome	ICC	95% CI	SEM	MD	MD% INT	MD% CON
vVT1 (km h^−1^)	0.250	− 0.153 to 0.582	1.52	4.2	7%	8%
hrVT1 (b min^−1^)	0.509	0.150 to 0.749	7.75	21.5	0%	0%
vVT2 (km h^−1^)	0.736	0.486 to 0.874	0.60	1.7	0%	8%
hrVT2 (b min^−1^)	0.664	0.372 to 0.542	5.11	14.2	0%	0%
$\dot{V}$O_2max/*M*_ (mL min^−1^ kg^−1^)	0.742	0.467 to 0.868	2.53	7.0	29%	8%
SystolicBP (mmHg)	0.471	0.110 to 0.723	7.71	21.4	14%	0%
DiastolicBP (mmHg)	0.471	0.110 to 0.726	4.77	12.2	14%	0%
PWV (m s^−1^)	0.490	0.134 to 0.734	0.25	0.7	7%	0%
restHR (b min^−1^)	0.706	0.110 to 7.29	5.90	16.5	7%	0%
RMSSD (ms)	0.742	0.467 to 0.868	2.53	7.0	0%	8%
SDNN (ms)	0.697	0.423 to 0.854	82.99	230.0	0%	0%

*ICC* intraclass correlation coefficient, *CI* confidence interval, *SEM* standard error of the measurement, *MD* minimal difference to be considered real, *MD%* percentage of participants with a difference ≥ MD, *INT* intervention group, *CON* control group, *vVT1* velocity at ventilatory threshold 1, *hrVT1* heart rate at ventilatory threshold 1, *vVT2* velocity at ventilatory threshold 2, *hrVT2* heart rate at ventilatory threshold 2, $\dot{V}$*O*_*2max/M*_ maximal oxygen consumption normalized per body mass, *sBP* systolic blood pressure, *dBP* diastolic blood pressure, *PWV* pulse wave velocity, *restHR* resting heart rate, *RMSSD* root mean square of successive RR interval differences, *SDNN* Standard Deviation of NN Interval

Individual patterns of training response﻿s for endurance and cardiovascular parameters following 8 weeks of running training are displayed in Fig. 2.

## Discussion

In this study, our primary objective was to assess the effects of two distinct training approaches—polarized training adapted to the MC and anti-MC-periodized training—on the endurance performance and cardiovascular parameters of moderately active females.

### Endurance performance

Both training programs significantly improved aerobic capacity and running velocity at the ventilatory thresholds. However, our findings revealed no significant differences between the two training approaches.

This indicates that, for moderately active females, adapting running training to the MC does not confer a substantial performance advantage. These results align with earlier findings from our research group (Kubica et al. 2023). A major limitation of the previous study was that a significant portion of the control group's training coincidentally aligned with the MC phases. In the current study, we therefore implemented a polarized training adapted contrary to the MC in the CON to increase the differences between the training protocols. Despite these adjustments in the study design, no differences were observed between the groups.

Unfortunately, limited research on the effect of MC-adapted endurance training makes it difficult to classify the current results further. However, previous research on resistance training indicates a positive effect of adapting the training to the MC (Kissow et al. 2022; Thompson et al. 2020). It has been suggested that metabolic shifts during the MC may alter training readiness and response (Devries 2016; Hackney 2021; Hackney et al. 2021; Isacco and Boisseau 2017; Vigh-Larsen et al. 2021). However, the current body of literature remains inconsistent (Hulton et al. 2021). The inconsistent results may be attributed to factors such as training intensity (Oosthuyse and Bosch 2010) and nutritional status (Hulton et al. 2021; McLay et al. 2007), which are often not adequately controlled in research studies. Those factors could potentially play a more influential role in metabolism and thus have a more substantial impact on training readiness and response than the MC. In the present study, we controlled the training intensity using HR. Unfortunately, the diet was not fully controlled. Participants were only encouraged to maintain adequate carbohydrate intake before each training session. Thus, it cannot be ruled out that differences in nutritional status before the exercises could have mitigated potential metabolic shifts associated with the MC (Hulton et al. 2021).

Apart from the energy metabolism itself, various other factors, including individual athlete perceptions of the MC's impact on training and performance, as well as the lived experiences and stigmas related to the MC, can also influence training readiness and responses (Carmichael et al. 2021; Kolić et al. 2021). Additionally, depending on the exercise intensity, psychological responses to training seem to be altered during the MC (Prado et al. 2021). Those alterations in motivation and affective response might impact training adherence and, therefore, long-term development (Prado et al. 2021). The complexity of overall performance dynamics (Coffey and Hawley 2007), the influence of psychological responses (Prado et al. 2021), compounded by individual variability in how MC phases may affect performance (Julian et al. 2017)*,* makes it crucial to consider individual changes.

Therefore, we examined individual responses using the MD. Notably, in the INT, 31% of females exhibited changes in $\dot{V}$O_2max*/M*_ that exceeded the MD, compared to 8% in the CON. This indicates that the training intervention adapted to the MC resulted in a higher responder rate. However, it is noteworthy that three-quarters of the responders from the INT group and half from the CON group were initially categorized as "unfit," with an initial $\dot{V}$O_2max*/M*_ < 37 ml/min/kg (Decroix et al. 2016). This finding implies a potential influence of the participants' initial fitness levels on the training response, which should be addressed in future studies with larger sample sizes. For all other endurance parameters, changes exceeding the MD were less common in both groups.

According to the literature, the presence of PMS in active females might also impact individual training responses and lead to MC-based performance changes (Carmichael et al. 2021). Therefore, a subgroup analysis of females with and without PMS is recommended to explore the potential effects of PMS and to identify responders and non-responders (Carmichael et al. 2021). However, a statistical sub-group analysis was not feasible due to the small sample size and lack of power. Still, we were not able to identify a pattern when looking at the MD for each outcome and participant and the influence of PMS (s. Fig. 2). Therefore, we assume that PMS had no impact on our results.

In summary, both training approaches led to significant improvements in aerobic capacity and running velocity at the ventilatory thresholds; however, the MC-adapted and non-adapted training did not yield discernible differences in specific performance parameters. Also, the individual responses underscore the complexity and individuality of performance responses to MC-adapted endurance training. In light of our non-significant findings and considering prior research on MC-adapted endurance training (Kubica et al. 2023), it seems plausible to suggest that MC-adapted training may not confer discernible performance benefits in healthy, moderately active, naturally menstruating, young adult females. Nevertheless, further investigation is needed to validate and refine this preliminary conclusion.

### Cardiovascular parameters

Besides endurance performance, our study investigated the impact of running training, adapted to and contrary to the MC, on various cardiovascular parameters. The results showed no significant interaction effects or time-related changes in all cardiovascular parameters.

To the best of our knowledge, limited research has explored the impact of MC-adapted endurance training on cardiovascular parameters. The current findings align with a previous investigation from our research group, which demonstrated that MC-adapted endurance training did not yield substantial benefits regarding cardiovascular parameters (Kubica et al. 2023).

When comparing the current results with research on the general influence of endurance training on cardiovascular parameters, the absence of time-related changes in our study contradicts with previous research (Cornelissen and Smart 2013; Reimers et al. 2018). Current meta-analyses indicate a positive effect of endurance training on cardiovascular parameters, specifically by a reduction in BP and restHR (Cornelissen and Smart 2013; Reimers et al. 2018). The recent meta-analyses point out that, on average, females experience a HR reduction of approximately 3.8 bpm following endurance exercise interventions (Reimers et al. 2018). Our results demonstrated comparable but non-significant changes in restHR, with an average decrease of 3.8 bpm in the INT and 3.7 bpm in the CON. However, it is essential to recognize that the intervention duration in our study falls on the lower end of the spectrum compared to the wide range of intervention durations in the meta-analysis (6–104 weeks) (Reimers et al. 2018). This may explain the lack of a significant effect on restHR in our study.

Also, no significant time-related effects were found for systolic BP and diastolic BP in our study. Previous research on the effect of MC-adapted endurance training on BP is limited. To our knowledge, only one previous study from our research group (Kubica et al. 2023) investigated the effects, with no effects of the MC-adapted endurance training as well as block-periodized endurance training led on BP. However, a meta-analysis by Cornelissen et al. (2013) reported that endurance training reduces systolic BP but not diastolic BP. Notably, the effects on systolic BP are more pronounced in individuals with prehypertension or hypertension. Given that our participant group was predominantly healthy, the non-significant changes in systolic BP may be attributed to their baseline health status (Cornelissen et al. 2013).

The PWV measures the velocity of the central pulse wave and represents a marker of arterial stiffness. Increased PWV is a predictive factor for cardiovascular events, even when considering other established risk factors (Ben-Shlomo et al. 2014). In our study, we were not able to detect any significant effects of the 8-week endurance training intervention on PWV. According to a meta-analysis, aerobic endurance training is generally associated with a significant decrease in the central PWV of − 0.67 m s^−1^ (Huang et al. 2016). Nevertheless, subgroup analyses in the meta-analysis indicate variations, with reduced effects among healthy individuals (weighted mean difference of − 0.19 m s^−1^), compared to those with cardiovascular diseases (weighted mean difference of − 0.55 m s^−1^), longer intervention durations (weighted mean difference 4–8 week interventions: − 0.35 m s^−1^ vs. > 16 weeks: − 1.19 m s^−1^), and more significant changes in $\dot{V}$O_2max*/M*_ (weighted mean difference change in $\dot{V}$O_2max_ ≤ 10% = − 0.40 m s^−1^ vs. ≥ 20% = − 1.72 m s^−1^), and in male compared to female participants (weighted mean difference males: − 0.50 m s^−1^ vs. females: − 0.36 m s^−1^) (Huang et al. 2016). The relatively short 8-week intervention duration in our study, coupled with the characteristics of our participants, may explain the limited effect of endurance training on PWV.

Also, no significant changes in the HRV indices could be detected in the INT or CON group. Even though it has been shown that regular endurance training can positively affect HRV parameters, its effectiveness in healthy young to middle-aged individuals is still subject to critical discussion (Dutra et al. 2013). The inclusion of only young and healthy individuals may account for the absence of significant results in the current study. Regarding the individual responses, only 14% of the participants in each group reached the MD.

### Limitations

Some limitations should be considered when interpreting the present results.

First, the MC was determined by calendar-based counting, daily basal body temperature and ovulation measurements due to financial limitations. Even though this is a practical and cost-effective approach to determining MC phases, accuracy is limited. This restricts further interpretations regarding hormone changes between responders and non-responders to the training program. Future studies should apply a three-step verification, including serum/plasma hormone analysis, to verify menstrual cycle phases and hormonal concentrations (Johnson et al. 2018; Schaumberg et al. 2017) and investigate the effects of individual hormone levels on the training responsiveness.

Second, we only included healthy participants with a regular MC to reduce the heterogeneity and following ambiguity in the results. However, moderately active, naturally menstruating females represent only part of society, as MC irregularities are highly prevalent among females worldwide (Gimunová et al. 2022; Righi and Barroso 2022). Further, results in a highly trained population where even minor biological differences are important might differ from our study population of moderately active females. This limits the overall generalizability of our results to a broader population, including less or more physically active females or females with menstrual irregularities or disorders.

Third, to ensure a comparable training load between the INT and CON, the training session distribution of two out of three MC phases was manipulated between the two groups, and one MC phase (early to mid-luteal phase) remained constant. Therefore, the overall impact was possibly too low to evoke differences between the MC-adapted and contrary-MC-adapted training, especially over the course of 8 weeks.

Finally, our study consisted of a relatively small sample size due to the exclusion of participants prior to the intervention and before the data analysis, as well as dropouts during the intervention, leading to a decreased statistical power. Based on a post-hoc analysis of our $\dot{V}$O_2max_ results, we conducted a required sample size analysis with G*Power 3.1. (Faul et al. 2007) for an ANOVA with repeated measures and within-between interaction. With a given power of 0.8 a total sample size *N* = 88 participants (*n* = 44 per group) would be required. Hence, it must be acknowledged that the study may have been underpowered.

However, the current study can contribute to future sample size estimations by providing results for power calculations. Future studies should also consider recruiting larger samples to compensate for possible dropouts for the non-fulfillment of inclusion criteria related to the MC (Elliott-Sale et al. 2021).

## Conclusion

In summary, based on our results, we conclude that among healthy, naturally menstruating females, an 8-week polarized running training program consisting of three weekly training sessions significantly enhances performance but has no significant effect on cardiovascular parameters. Furthermore, our study did not reveal any discernible differences in performance and cardiovascular parameters between MC-adapted and contrary-MC-adapted training approaches. This contradicts previous studies on MC-based resistance training reporting positive effects on various performance parameters but aligns with prior research on endurance training. Future studies with an extended intervention period, a larger sample size, and a more reliable MC determination are warranted to advance our understanding of the influence of training adaptation in relation to the MC phase. Based on our findings, it appears that MC-adapted training may not provide discernible performance benefits in moderately trained women. However, this observation does not diminish the importance of considering cycle phases in training planning. The heightened responder rate underscores the significant individual variability in how the MC influences training outcomes and physiological adaptations. Therefore, it is prudent for athletes and trainers to adopt a highly personalized approach when addressing this matter.

In future investigations, several methodological changes and research avenues could be explored. These include alterations in the training intervention, involving variations in training frequency with a focus on high-intensity interval training or moderate intensity continuous training. It could also entail considering other outcomes such as wellbeing and enjoyment, particularly among recreationally active females. Further, investigating whether adapting training to MC phases yields differential effects in elite athletes or among females with MC irregularities could provide valuable insights. Also, we would recommend a prolonged intervention duration, at least three or more MCs.

## Data Availability

The data presented in this study are available on request from the corresponding author.

## Abbreviations

ANOVA: Analysis of variance
BMI: Body mass index
BP: Blood pressure
CON: Control group
*d*: Cohen's *d*
HR: Heart rate
HRV: Heart rate variability
ICC: Intraclass correlation coefficient
INT: Intervention group
MC: Menstrual cycle
MD: Mean difference to be considered real
*η*_p_^2^: Partial eta squared
PWV: Pulse wave velocity
restHR: Resting heart rate
RMSSD: Root mean square of successive RR interval differences
RPE: Rate of perceived exertion
SDNN: Standard deviation of NN intervals
SEM: Standard error of measurement
*v*: Velocity
$\dot{V}$O_2_max: Maximal oxygen uptake
VT1: Ventilatory threshold 1
VT2: Ventilatory threshold 2
WHtR: Waist-to-height ratio
